# Rac1 is dispensable for oocyte maturation and female fertility in vivo

**DOI:** 10.1371/journal.pone.0177202

**Published:** 2017-05-18

**Authors:** Jian-Xiu Hao, Tie-Gang Meng, Li-Hua Fan, Yuan-Qing Yao

**Affiliations:** 1 Department of Obstetrics and Gynecology, General Hospital of Chinese People’s Liberation Army, Beijing, China; 2 State Key Laboratory of Stem Cell and Reproductive Biology, Institute of Zoology, Chinese Academy of Sciences, Beijing, China; 3 University of Chinese Academy of Sciences, Beijing, China; Nanjing Agricultural University, CHINA

## Abstract

Oocyte maturation, the important process to produce female haploid gamete, accompanies with polarity establishment and highly asymmetric cell division to emit minor polar body within little cytoplasm. Microfilaments play central roles in polarity establishment and asymmetric cell division. Several actin regulators like WASP protein family as well as small GTPases function in microfilament dynamics, involving the process. Rac1, one member of RhoGTPases, has been reported to regulate the polarity and asymmetric cell division in mouse oocytes in vitro. The physiological role of Rac1 in mouse oocyte remains unknown. By conditional knockout technology, we specifically deleted *Rac1* gene in mouse oocyte, and found that Rac1 deletion exerted little effect on mouse oocyte maturation including polarity establishment and asymmetric division, and the mutant mice showed normal fertility.

## Introduction

In most mammalian ovaries, oocytes are arrested at diplotene in prophase for as long as several decades, which are characteristic of the germinal vesicle (GV). Following the luteinizing hormone (LH) surge, oocytes resume meiosis, manifested by germinal vesicle breakdown (GVBD). After GVBD, the microtubules organize into the Metaphase I (MI) spindle around the chromosomes at about the center of the oocyte, then the MI spindle migrates to the cortex randomly[1], followed by homologous chromosomes segregation and the first polar body emission. Then the metaphase II spindle forms, parallel to the cortex, indicting the mature MII oocytes produced, awaiting fertilization during which the sister chromatids separate and the second polar body emits [2]. During oocyte maturation and fertilization, two important events occur in nucleus and cytoplasm respectively. For nucleus, the homologous chromosomes and sister chromatids segregate during the two meiosis respectively, producing the haploid female gamete. For cytoplasm, the polar bodies formed in the two meiosis are very small, containing little cytoplasm. Importantly, the two events occur in nucleus and cytoplasm respectively linked by the asymmetric spindle migration as well as the polarity establishment.

Recent studies have greatly promoted the knowledge of mechanisms underlying oocyte polarity and asymmetric division. Microfilaments play central roles in polarity establishment and asymmetric cell division in mouse oocytes [3; 4]. Studies show that actin nucleators Formin 2 [5; 6; 7], Spire1/2 [8; 9] and Arp2/3 protein complex [10; 11; 12] regulates asymmetric spindle positioning, polarity formation as well as polar body emission respectively or cooperatively in mouse oocytes. Upstream of the actin nucleators, some nucleation promoting factors (NPFs) and Rho-GTPases could mediate their activities to affect the process of spindle migration and polarity establishment during oocyte maturation.

Rho-GTPases, one subfamily of the small GTPases, consist of RhoA, Cdc42 and Rac1. It is widely reported that the three GTPases play fundamental functions in polarity and asymmetric cell division in oocytes. RhoA regulates contractile ring formation during polar body emission in both *Xenopus* oocytes [13] and mouse oocytes [14]. Cdc42 inhibits polar body emission by disrupting actin cap formation through actin nucleator Arp2/3 protein complex [15]. For Rac1, it was reported that Rac1 inhibition caused MI spindle elongation and MII spindle detachment form the cortex in vitro [16]. It is worth noting that there exists large difference between the data obtained from in vivo and in vitro studies on the functions of Cdc42 [15]. So the physiological roles of Rac1 need to be addressed. By conditional knockout technology, we specifically deleted *Rac1* in oocyte from primary follicle and later. We found that *Rac1* deletion has no effect on oocyte maturation and female fertility. In details, during *Rac1*^*flox/flox*^; *ZGre*^*+*^ oocyte maturation, the important events including spindle organization, migration, homologous chromosomes segregation, polarity and polar body emission are normally occurring. Considering the similarity of Rac1 and Rac3 both expressed in mouse oocytes, we carried out the Rac3 knockdown experiment in Rac1-deleted oocytes. We found that knockdown of Rac3 in Rac1-deleted oocytes yet exerted little effect on oocyte maturation.

## Results

### Generation of mutant mice with oocyte-specific deletion of *Rac1*

**Fig 1:**
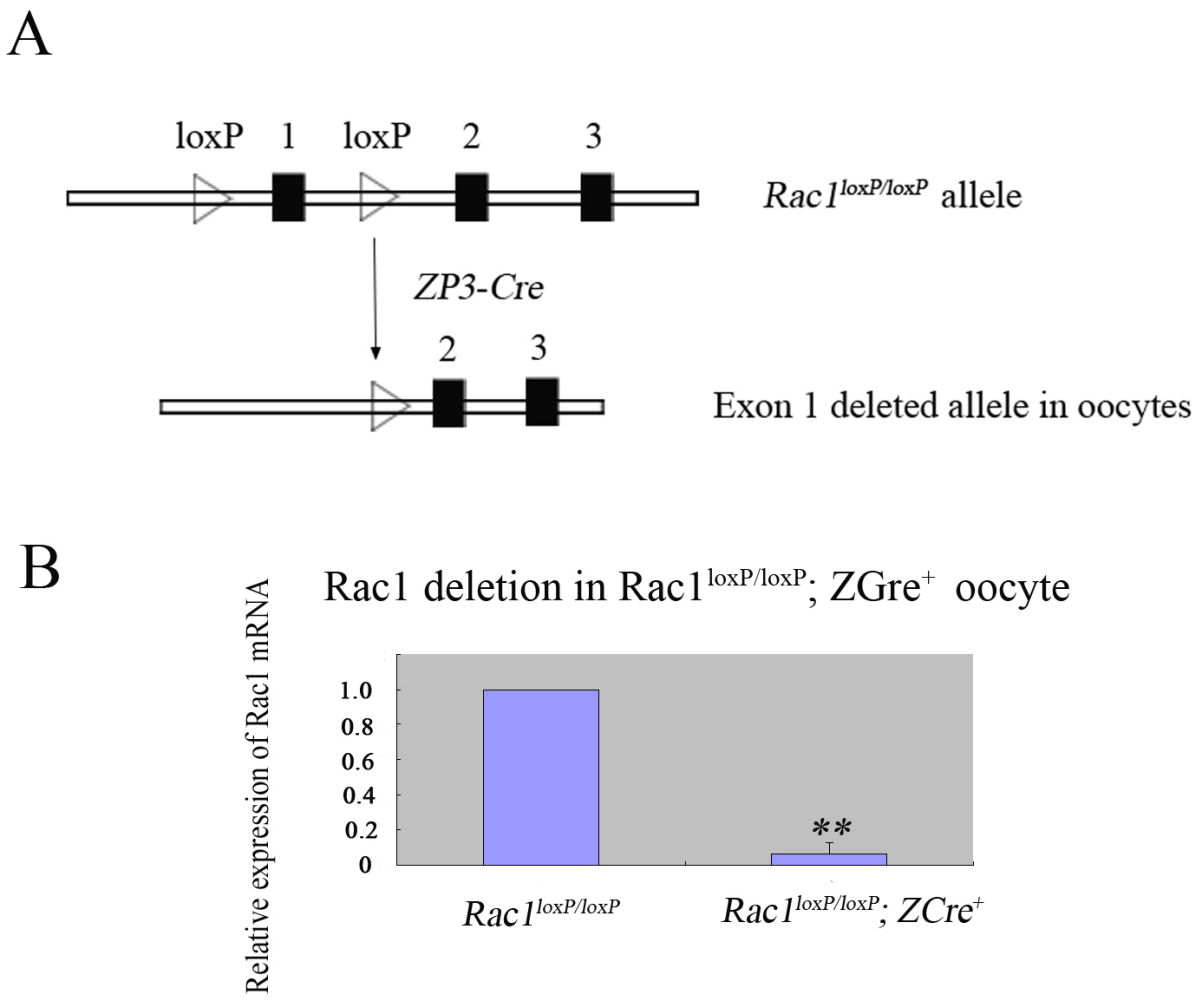
Specific deletion of *Rac1* in mouse oocyte. (A) Schematic representation for deletion of *Rac1* exon 1 by *ZP3-Cre*-mediated recombinase in oocytes. (B) Quantificational real-time PCR analysis showing the Rac1 deletion in oocytes of *Rac1*^*loxP/loxP*^; *ZCre*^*+*^ mice. The GV oocytes from *Rac1*^*loxP/loxP*^; *ZCre*^*+*^ and *Rac1*^*loxP/loxP*^ mice were used for RNA isolation and quantitative RT-PCR. The experiments were repeated three times. ** P<0.01.

To explore the physiological role of Rac1 in oocyte, we generated mice in which the *Rac1* gene was deleted in oocytes of growing follicles. The mutant mice (referred to as *Rac1*^*loxP/loxP*^; *ZCre*^*+*^ mice) was generated by crossing *Rac1*^*flox/flox*^ mice with the transgenic mice expressing *zona pellucida 3 (ZP3)*-promoter-mediated Cre recombinase (Fig 1A). By quantitative real-time PCR, we found that the expression of *Rac1* mRNA was greatly decreased in oocytes of mutant mice (Fig 1B), showing successful deletion of the *Rac1* gene from oocytes.

### Normal fertility and MII eggs in *Rac1*^*loxP/loxP*^; *ZCre*^*+*^ mice

**Fig 2:**
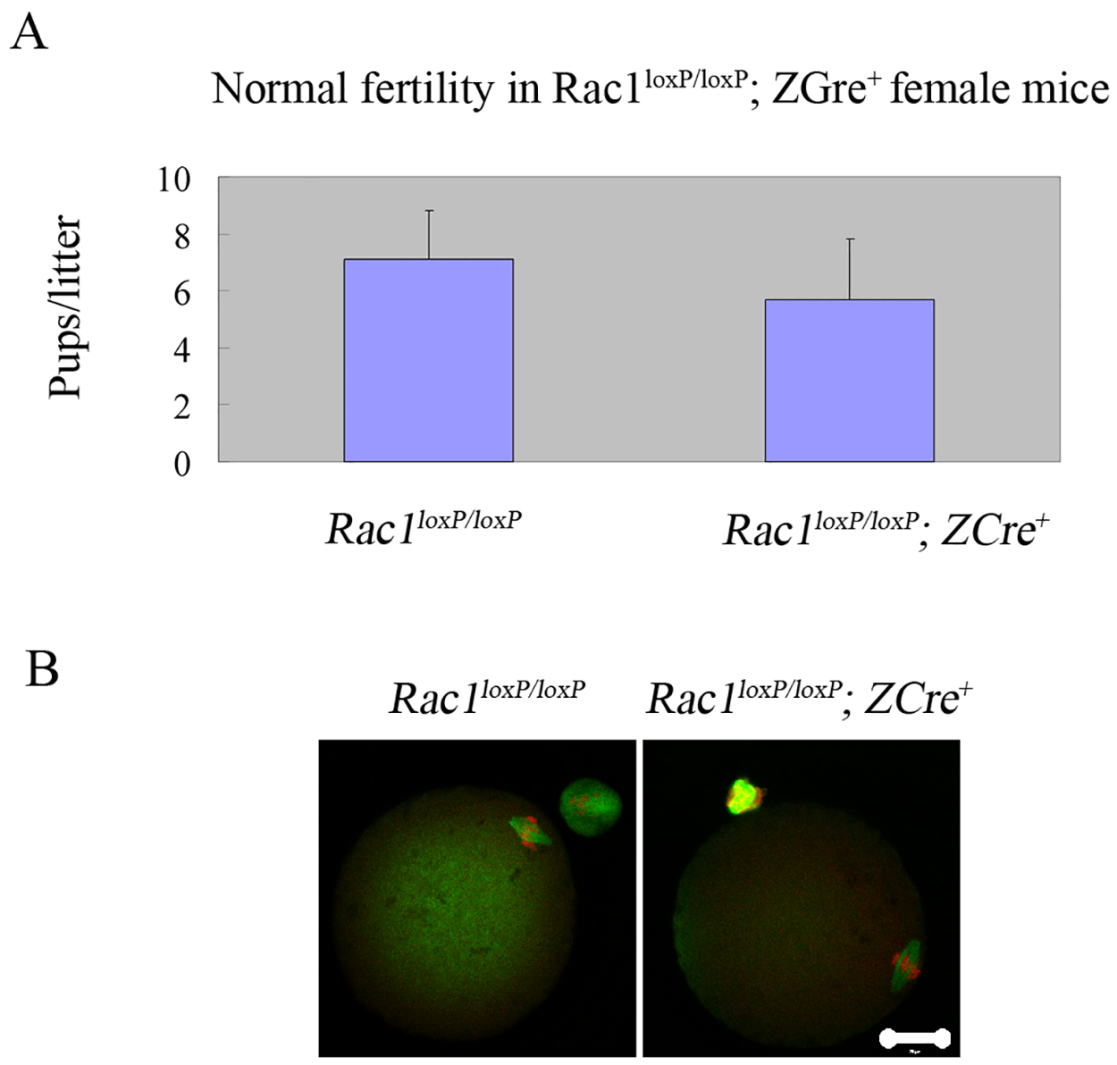
Normal fertility and MII eggs in female mice with oocyte-specific deletion of *Rac1*. (A) Comparison of the average number of pups per litter per female mice during a period of 6 months between *Rac1*^*loxP/loxP*^; *ZCre*^*+*^ (n = 4) and *Rac1*^*loxP/loxP*^ mice (n = 3). P>0.05. The data are mean ± SEM. (B) Normal spindle/chromosomes in *Rac1*^*loxP/loxP*^; *ZCre*^*+*^ MII eggs. Ovulated eggss were fixed and stained with anti-α-tubulin- FITC antibody (green) and counterstained with PI to visualize DNA (red). Experiments were repeated at least three times and representative images were shown.

We carried out the experiment of fertility test first after we got the mutant mice. We mated the *Rac1*^*loxP/loxP*^; *ZCre*^*+*^ and *Rac1*^*loxP/loxP*^ mice with fertility-proven male mice respectively for six months. As is shown in Fig 2A, there is no significant difference in fertility between *Rac1*^*loxP/loxP*^; *ZCre*^*+*^ and *Rac1*^*loxP/loxP*^ mice. Then we checked the MII eggs from both the two groups. As expected, *Rac1*^*loxP/loxP*^; *ZCre*^*+*^ mice produce normal MII eggs (Fig 2B).

### *Rac1* deletion exerts little effect on oocyte maturation and polarity formation

**Fig 3:**
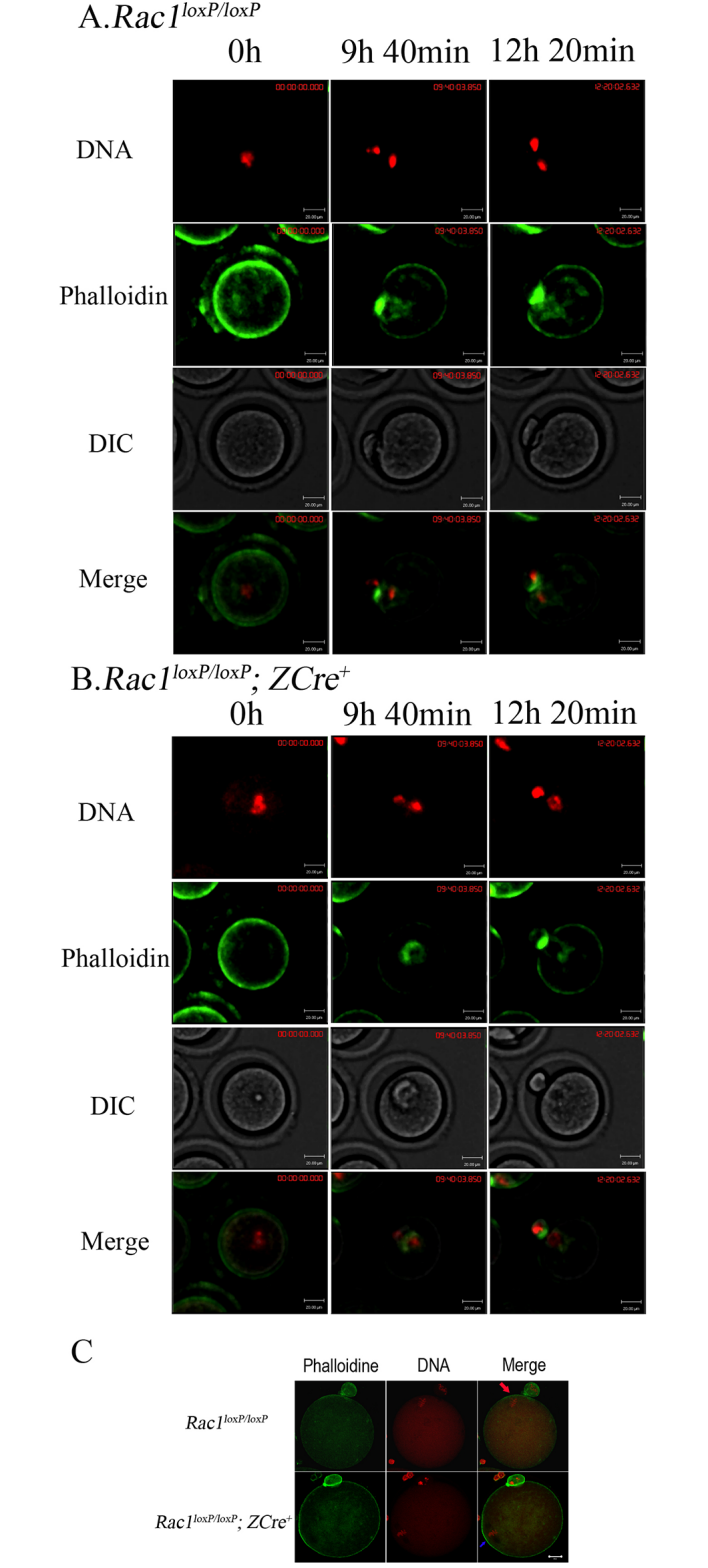
*Rac1* deletion has no effect on oocyte maturation. For A and B, chromosomes were stained in blue by Hoechst 33342 (2ng/mL in M16 medium) and the blue was changed into red. Alexa 488-phalloidin was microinjected into GV oocytes to show the actin dynamics during oocyte maturation. Bar = 20 μM. (A) The dynamics of Alexa 488-phalloidin during *Rac1*^*loxP/loxP*^ oocyte maturation. (B) The dynamics of Alexa 488- phalloidin during *Rac1*^*loxP/loxP*^; *ZCre*^*+*^ maturation. (C) Normal actin cap in *rac1*^*loxP/loxP*^; *ZCre*^*+*^ MII eggs. Oocytes were stained Alexa 488-phalloidine (green) and PI (10μg/mL, red), and viewed with confocal microscopy. Bar = 20μM.

Though normal MII eggs produced in *Rac1*^*loxP/loxP*^; *ZCre*^*+*^ mice, it is still necessary to show if the process of oocyte maturation is normal. So we carried out the experiments of live oocyte imaging to show the process of oocyte maturation of *Rac1*^*loxP/loxP*^; *ZCre*^*+*^ and *Rac1*^*loxP/loxP*^ mice respectively by tracking the dynamics of actin (Alexa 488- phalloidin) and chromosomes. As shown in Fig 3A (also see S1 Movie), the *Rac1*^*loxP/loxP*^ oocytes underwent a normal meiotic maturation process. At 9h 40min, The homologous chromosomes segregated and the first polar body was emitted. After polar body formation, the MII chromosomes underneath the membrane and oocyte polarity (actin cap) were maintained. The *Rac1*^*loxP/loxP*^; *ZCre*^*+*^ oocytes underwent maturation normally and similar to the control oocytes (Fig 3B and S2 Movie). We further carried out immunofluorescent staining experiments to show that the actin cap remained intact in the fixed *Rac1*^*loxP/loxP*^; *ZCre*^*+*^ MII oocytes (Fig 3C).

### Rac3 depletion in *Rac1*^*loxP/loxP*^; *ZCre*^*+*^ oocytes has no effect on meiotic maturation

**Fig 4:**
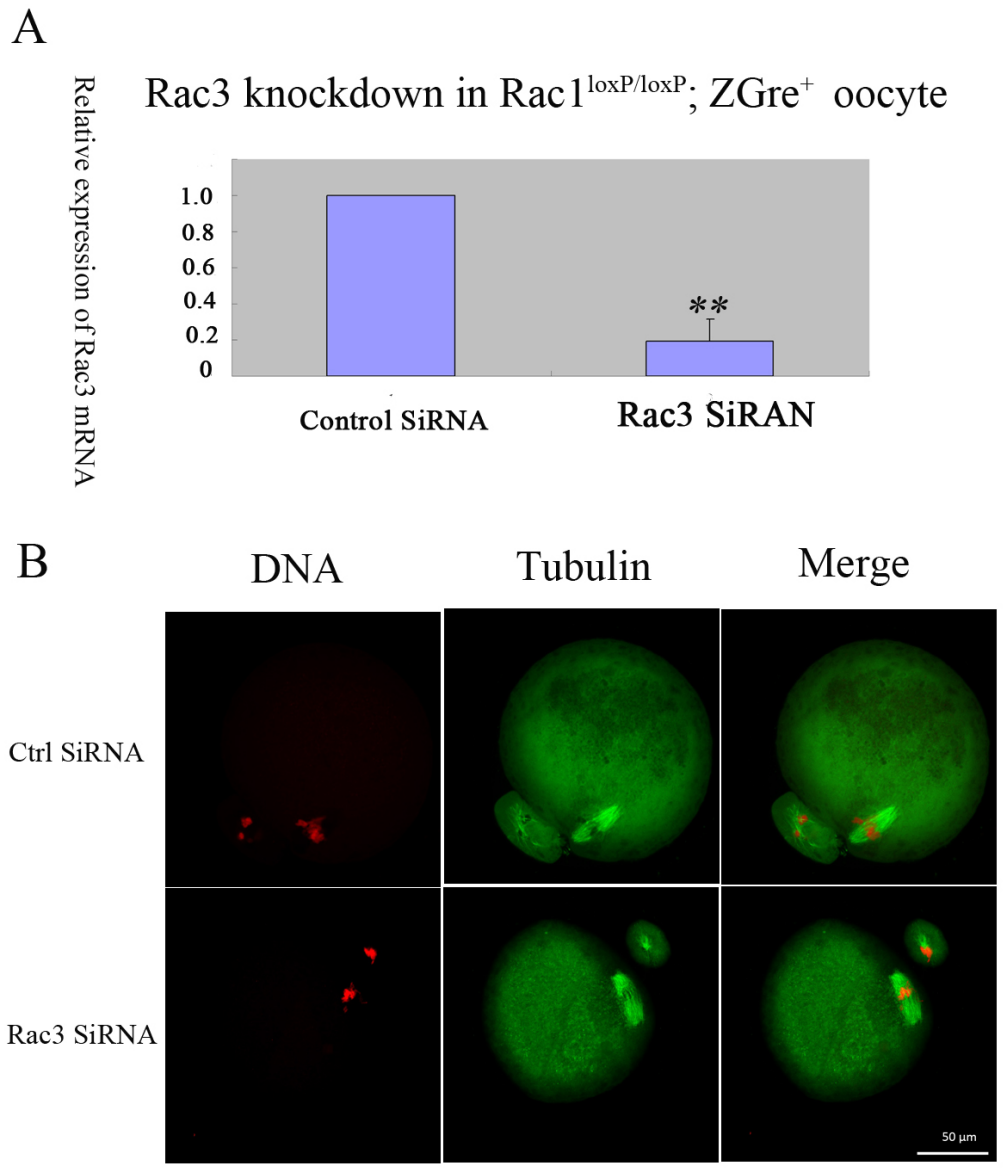
Knockdown of Rac3 has no effect on *Rac1*^*loxP/loxP*^; *ZCre*^*+*^ oocyte maturation. (A) Knockdown of Rac3 through RNA interference in *Rac1*^*loxP/loxP*^; *ZCre*^*+*^ oocytes. The *Rac1*^*loxP/loxP*^; *ZCre*^*+*^ oocytes were microinjected control SiRNA or Rac3 SiRNA, incubated in M16 medium with 2.5μM Milrinone for 24 hours, and then were collected for qRT-PCR. The experiments were repeated at least three times. ** P<0.01. (B) Normal MII eggs matured from Rac3-knockdowned- *Rac1*^*loxP/loxP*^; *ZCre*^*+*^ oocytes. The control SiRNA or Rac3 SiRNA was microinjected into *Rac1*^*loxP/loxP*^; *ZCre*^*+*^ oocytes. The oocytes were incubated in M16 medium with 2.5μM Milrinone for 24 hours, then were cultured in M16 medium, and the MII eggs were collected for immunofluorescent staining. The experiments were repeated for three times, and the typical images were shown. Green, spindle; Red,chromosomes.Bar,50μM.

The Rac protein family consists of three members-Rac1, Rac2 and Rac3.Rac2 mainly expresses in hematopoietic cells, and Rac1 and Rac3 express ubiquitously [17]. Rac1 and Rac3 share high similarity in sequence, and they might compensate for each other in function. We carried out Rac3 knockdown experiments to check the possibility that Rac3 compensate the functions of Rac1 in oocytes. As shown in Fig 4A, Rac3 could be knockdowned efficiently. We could see that knockdown of Rac3 in *Rac1*^*loxP/loxP*^; *ZCre*^*+*^ oocytes exerted little effect on oocyte maturation (Fig 4B).

## Discussion

By conditional knockout technology, we generated the mutant mice and studied the functions of Rac1 in mouse oocytes in vivo. Different from its roles obtained from the in vitro study, we found that Rac1 is indispensable for oocyte maturation and female fertility in mice. We found that Rac1 absence has little effect on the process of oocyte maturation including polarity formation and asymmetric cell division.

Microfilament regulators including WASP family protein and RhoGTPases play pivotal roles in polarity establishment, maintenance and asymmetric division during oocyte maturation. Among the related studies, different results were obtained from in vitro and in vivo studies. It is reported that N-WASP depletion resulted in polarity impairment in MII eggs [11], while oocyte-deletion of *N-WASP* showed that N-WASP is not required for polarity in MII eggs, but for cytokinesis completion [18]. For Cdc42, it had been reported that Cdc42 disruption causes spindle defects, impairs asymmetric spindle migration to the cortex and inhibits homologous chromosome segregation in *in vitro* study [19]. However, oocyte-specific deletion of *Cdc42 in vivo* does not impair normal bipolar spindle formation, asymmetric spindle positioning near the cortex, while it functions in polarity establishment and the first polar body emission [15]. For the reason, we tried to explore the physiological roles of Rac1. In in vitro study, it is found that Rac inhibition resulted in prometaphase I block and spindle elongation during oocyte maturation. In our study, we found that sole Rac1 knockout or Rac1 knockout with Rac3 Knockdown has little effect on the process of oocyte maturation. Oocyte-specific knockout of both *Rac1* and *Rac3* is the better way to solve the question.

## Materials and methods

All chemicals were purchased from Sigma (St. Louis, MO, USA) unless otherwise indicated.

### Animal

Homozygous *Rac1*^*loxP/loxP*^ mice (mixed background of 129S4/SvJae and BALB/c) were crossed with *Zp3-Cre* transgenic mice (C57BL/6J) to generate *Rac1*^*loxP/loxP*^; *ZCre*^*+*^ animals. Control mice were littermates possessing two *loxP* flanked alleles without Cre (*Rac1*^*loxP/loxP*^). Mice were maintained in alternating 12-hour light/dark cycles. Animal care and use were carried out in accordance with the Animal Research Committee guidelines, the study was approved by the animal ethics committee at PLA General Hospital.

### Oocyte collection and culture

Four-to-eight-week-old female mice were used in the experiments. For *in vitro* maturation, GV oocytes were collected and cultured in M16 medium under liquid paraffin oil at 37°C in an atmosphere of 5% CO_2_ in air. To obtain MII oocytes, mice were induced to superovulate by injection of 8 IU PMSG followed 48 h later by injection of 8 IU hCG. Fifteen hours after hCG injection, mice were sacrificed and the oviductal ampullae were broken to release the cumulus–oocyte complexes. MII oocytes were freed of cumulus cells by exposure to 300 μg/ml hyaluronidase. Oocytes were collected for immunofluorescent staining or microinjection.

### Microinjection, Rac3 knockdown and live oocyte imaging

Microinjection of Rac3 SiRNAs (Gene Pharma, Shanghai, China) or Alexa 488-phalloidin (Invitrogen, Carlsbad, CA, USA) into GV oocytes was performed using a Nikon Diaphot ECLIPSE TE 300 (Nikon UK Ltd., Kingston upon Thames, Surrey, UK) inverted microscope equipped with Narishige MM0202N hydraulic three-dimensional micromanipulators (Narishige Inc., Sea Cliff, NY, USA) and completed within 30 minutes.

Rac3 SiRNAs were microinjected into GV oocyte to knockdown Rac3. The subsequent sequences of Rac3 siRNAs were used at 20 μM each, Rac3 siRNA-1: 5’- GGAAGACUACGAUAGGCUUTT-3’;

Rac3 siRNA-2:5’- GCAAGAAGUGCACUGUAUUTT-3’;

Rac3 siRNA-3: 5’—GCCUUCCCAGGAGAAUAUATT-3’.

The same amount of negative control siRNAs were injected as control. Microinjected oocytes were cultured in M16 medium with 100μM IBMhX for 24 h. Then the oocytes were cultured in M16 medium.

For live oocyte imaging, after 1–2 hours of culture, the microinjected oocytes cultured in M16 medium with 10ng/mL Hoechst 33342 were used for live oocyte imaging on a Perkin Elmer precisely Ultra VIEW VOX confocal Imaging System (PerkinElmer, Waltham, MA, USA).

### Immunofluorescencent staining and confocal microscopy

For single staining of α-tubulin or actin, oocytes were fixed in 4% paraformaldehyde in phosphate-buffered saline (PBS, pH 7.4) for at least 30 min at room temperature. After being permeabilized with 0.5% Triton X-100 at room temperature for 20 min, oocytes were blocked in 1% bovine serum albumin-supplemented PBS for 1 h and incubated overnight at 4°C with 1:200 anti-α-tubulin- FITC antibody or 1:200 Alexa 488-phalloidin. After three washes in PBS containing 0.1% Tween-20 and 0.01% Triton X-100, the oocytes were stained with propidium iodide (PI, 10 μg/ml in PBS) or diamidino-phenyl-indole (DAPI, 10 μg/ml in PBS). Then, the oocytes were mounted on glass slides and examined with a confocal laser scanning microscope (Zeiss LSM 780, Germany).

### RNA isolation and quantitative real-time PCR analysis

Total RNA from GV oocytes was extracted using RNeasy micro purification kit (Qiagen; Hilden, Germany). Single-stranded cDNAs were generated with cDNA synthesis kit (Invitrogen; Carlsbad, CA, USA). Real-time PCR was conducted by using SYBR Premix Ex TaqTM kit (TaKaRa Biotechnology (Dalian) Co., Ltd., Japan) in ABI prism 7500 Sequence Detection System. Analysis of relative gene expression was measured by quantitative real-time PCR and the 2^−ΔΔCT^ Method.

## Statistics

All percentages from at least three repeated experiments were expressed as mean ± SEM. Data were analyzed by paired-samples t-test. P < 0.05 was considered statistically significant.

## Supporting information

S1 Checklist The ARRIVE guidelines checklist. (PDF)

S1 Movie Dynamics of chromosomes and Alexa 488-phalloidin during in vitro maturation of oocytes from Rac1loxP/loxP female mice, in accordance with the images shown in Fig 3A. (MOV)

S2 Movie Dynamics of chromosomes and Alexa 488-phalloidin during in vitro maturation of oocytes from Rac1loxP/loxP; ZCre+ female mice, in accordance with the pictures shown in Fig 3B. (MOV)
